# On Improvements in the Medical Art

**Published:** 1804-04-01

**Authors:** Julius Acer

**Affiliations:** London


### *To the Editors of the Medical and Phyſical Journal.*

Gentlemen,

YOUR popular Journal of medical intelligence seems to be a fit medium for suggesting improvements in the Medical Art. A late unfortunate duel near the metropolis, has given rise to the following observations and proposals.—Without supposing any deficiency in skill and energy attaching to the professional gentlemen who attended the case alluded to, and without any documents of greater authenticity than the medical evidence given before the Coroner's Jury, and that of a gentleman not in the profession, I have ventured to submit these thoughts to the profession and to the public.

According to the evidence already mentioned, this nobleman received a wound from a pistol bullet, which penetrated the right side of the chest and lodged in or about the medulla spinalis. From the described course of the ball it must have penetrated the outer and posterior part of the the lungs only, where the pulmonary arteries and veins are of smaller diameter than in the thicker part or at the root of the lungs. Had the ball however passed through the larger blood-vessels of the lungs, such wound was not inevitably mortal, because numerous examples of recovery under such circumstances have occurred. The wound of the lungs was not therefore the fatal sign which produced the surgical despair on this occasion. But the ball penetrated through or between some of the vertebræ about the middle of the back, and the sure sign of injury done to the medulla spinalis, viz. loss of sensation and motion in the lower extremities, followed. Was this loss of sensation and motion immediate upon receiving the wound, or did it progressively increase? This was the critical symptom, for upon this and its attendant effects does it appear that life or death depended, as well as the possible chance of relief by a surgical operation. Now the osseus canal for containing the medulla spinalis in the dorsal vertebræ is about eight-tenths of an inch in its transverse diameter, and seven-tenths of an inch between the anterior and posterior sides of it, and the ball of a modern duelling pistol is not more than four-tenths of an inch in diameter; it is therefore evident that the ball could not of itself wholly divide the spinal medulla, even supposing it to pass directly through the centre of the vertebral cavity. Other chances would arise in the mind of a surgeon, such as those of protruded portions of bone; of the ball lying within the theca vertebralis, without having divided any part of the medulla, and all the degrees and combinations of these circumstances. Whether or not any external marks or sensations on the region of the spine indicated the place of the ball's lodgement, I am uninformed, but it is most probable there were; and if not, the course of the ball would have directed a reasonable guess at the precise part. To propose any surgical operation for the relief of such danger is not very consistent with professional prudence, but it may be a question with many persons, whether such prudence ought to decide the fate of a man, whose rank and talents promised (when duly regulated) the highest services to his country. It is said, upon good authority, that large doses of opium, with a view to smooth the way to death, was the only professional treatment adopted in this instance; and as these observations are not levelled at the respectable gentlemen employed, but are addressed to the profession for future occasions, and to the public, as an encourageencouragement to support a desperate effort rather than abandon life without giving a chance, I feel warranted in the freedom of my argument. It has happened in my experience, that the exhibition of opium increases symptomatic fever, and promotes hæmorrhages from wounds; which last I suppose to ensue principally from its diminishing the contractile powers of the blood vessels, and perhaps also the coagulating propensity of the blood.—Opium therefore, on these principles, would hasten death by loading the lungs and the cavity of the chest with blood, whilst the other wound in the spine continued its natural progress.

As a faint hope, and with a sincere wish to promote the utility and rank of the profession, I would propose to treat a future similar case in the following manner. To bleed freely from the arm as soon as the mind had recovered the shock, and to keep the pulse constantly low and feeble during the first six or seven days, by repeating the operation according to the pulse, and by a strict abstinence from nutritious and stimulating food. If the injured part of the spine can be discerned, or fairly indicated, then I see no objection to an operation similar to trepanning on the skull, by the help of small saws, such as described in Mr. Hey's Surgery. This operation could be put in practice without any immediate ill consequence, because after the integuments and muscles were removed, and the two rami of the spinous process sawed through, the canal of the spine would be exposed, so as to enable the surgeon to remove any extraneous or injurious body, and at least a chance be given to the patient not much worse than that afforded by trepanning the skull. Whether such an attempt was or was not warrantable in the case now spoken of, I cannot tell; but I trust the known rank of these professional gentlemen would induce them to exert every possible effort, and even to risk their own prudential feelings for such a grand object. I am, &c.

*London, March* 19, 1804. JULIUS ACER.

